# Is MPP a good prognostic factor in stage III lung adenocarcinoma with EGFR exon 19 mutation?

**DOI:** 10.18632/oncotarget.16505

**Published:** 2017-03-23

**Authors:** Tian Zhang, Jing Wang, Yanjun Su, Xi Chen, Qingna Yan, Qi Li, Leina Sun, Yuwen Wang, Puchun Er, Qingsong Pang, Ping Wang

**Affiliations:** ^1^ Department of Radiation Oncology, Key Laboratory of Cancer Prevention and Therapy, National Clinical Research Centre of Cancer, Tianjin Medical University Cancer Institute and Hospital, Tianjin, China; ^2^ Department of Lung Cancer, Tianjin Medical University Cancer Institute and Hospital, Tianjin, China; ^3^ Department of Pathology, Tianjin Medical University Cancer Institute and Hospital, Tianjin, China

**Keywords:** lung adenocarcinoma, classic EGFR mutations, micropapillary pattern, tyrosine kinase inhibitors

## Abstract

Epidermal growth factor receptor (EGFR) is a transmembrane glycoprotein encoded by a gene located in the short arm of chromosome 7. This study aimed to investigate the clinicopathologic characteristics of classic EGFR exon mutation in Chinese patients with TMN stage III lung adenocarcinoma who received radical surgery. A total of 1,801 lung adenocarcinomas were analyzed for mutations in EGFR; 35% exhibited mutation of classic EGFR exons. Clinical and pathologic characteristics of patients with EGFR exon 19 mutation were compared with those who harbored EGFR exon 21 mutation. Patients with EGFR exon 19 mutation had a higher overall survival (OS, p=0.023) than those harboring EGFR exon 21 mutation. Our results demonstrated that patients with a micropapillary pattern (MPP) pathologic type in EGFR exon 19 mutation had a higher OS (p=0.022), and patients with exon 19 mutation were more sensitive to EGFR–tyrosine kinase inhibitors (p=0.032). The results of the current study can be used in decision-making regarding the treatment of patients with classic EGFR exon mutations.

## INTRODUCTION

Lung cancer is the most frequent cause of cancer-related death worldwide, with non-small cell lung cancer (NSCLC) being the most common type [1, 2]. Improved understanding of genetic alteration in lung cancer has led to the development of many onco-targeted drugs and significant achievements [3–5].

Activating mutations of epidermal growth factor receptor (EGFR) are identified in about 20% of lung adenocarcinomas in Western countries [6] and 40%–60% of lung adenocarcinomas in East Asia [7–9]. These mutations, which mainly consist of EGFR exon 19 deletion (~50%) and exon 21 L858R mutation (~40%), are highly responsive to EGFR–tyrosine kinase inhibitors (EGFR–TKIs), such as gefitinib and erlotinib [4, 10, 11]. However, for stage III patients with EGFR mutations who received radical surgery, the adjuvant therapy that provides better results remains unclear.

As a unique pathological morphology, the micropapillary pattern (MPP) has drawn increasing attention in recent years. The micropapillary structure, which has been described as highly invasive and metastatic, is predictive of poor prognosis. Meanwhile, the suitability of the result for EGFR mutation remains unclear, and the prognostic value of MPP remains inconclusive in advanced-stage lung adenocarcinoma.

In the present study, we retrospectively investigated the clinicopathologic characteristics and prognosis of patients with activating EGFR exon mutations in a large cohort of patients with lung adenocarcinoma. We found that patients with exon 19 and the MPP pathological type had longer overall survival (OS), compared with those harboring exon 21 mutation or the non-MPP pathological type; in addition, patients with exon 19 mutation exhibited a better response to EGFR–TKIs, compared with patients with exon 21 mutation.

## RESULTS

A total of 1,801 patients with lung adenocarcinoma diagnosed from January 2011 to December 2014 were screened for EGFR mutation status. Among these patients, 678 (37.6%) harbored mutations in EGFR; of this number, 636 (93.8% of 678) cases with classic activating mutations (exon 19 or exon 21 mutations) and 42 (6.2% of 678) cases with rare mutations (exon 18 or exon 20 mutations) were detected.

**Figure 1:**
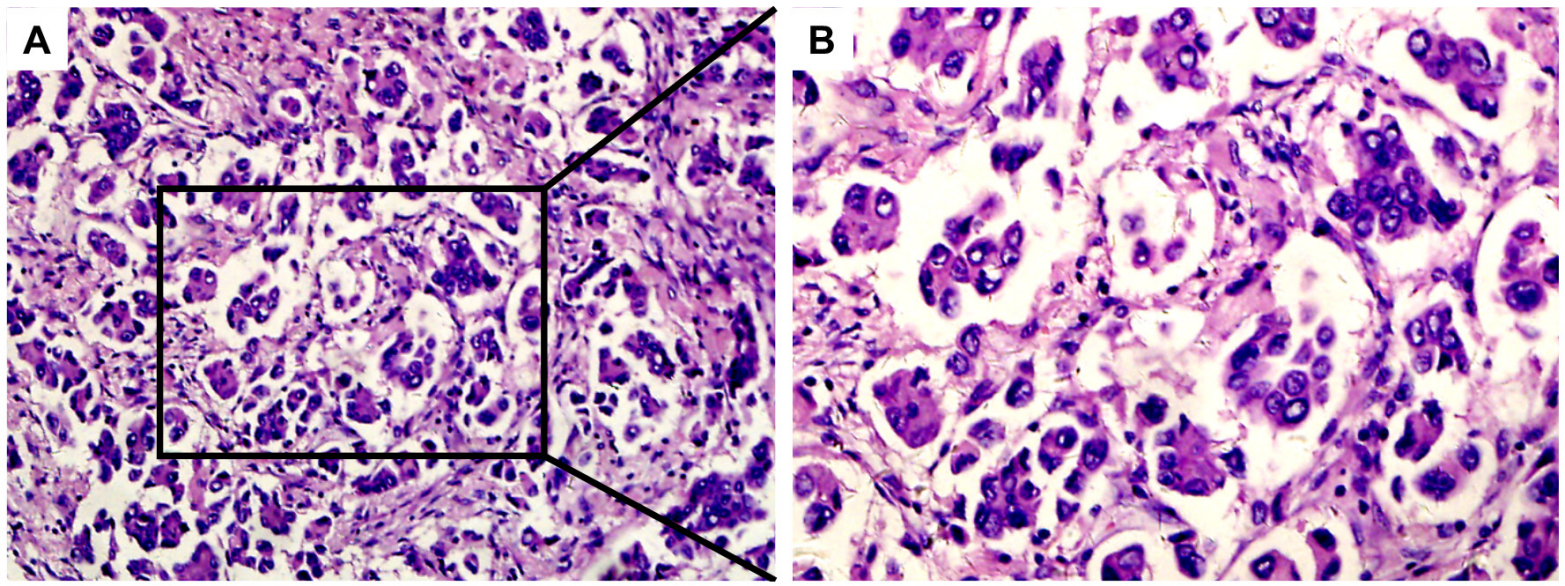
Hematoxylin–eosin staining of MPP-positive specimens MPP-predominant specimen (**A**, ×100 magnification; **B**, ×200 magnification).

Of the 636 patients with activating mutations of EGFR exon, 168 were tumor-node-metastasis (TNM) stage III cases who received radical surgery. These patients had a median follow-up duration of 30 months (range: 4–61 months). Of the 168 cases, 79 (47.02%) were carrying EGFR exon 19 mutations, 65 (38.7%) were over 60 years old, and 109 (64.9%) were never-smokers. The predominant pathological subtype included 89 (53.0%) cases with MPP (Figure 1). No significant differences were found between the patients carrying EGFR exon 19 mutation and those with EGFR exon 21 mutation with respect to gender, age, smoking history, Karnofsky Performance Status (KPS) score, TNM stage, and pathological types (Table 1).

**Table 1:** Comparison of clinical characteristics between NSCLCs harboring EGFR exon 19 and EGFR exon 21 mutation

Characteristics	Total	Exon 19	Exon 21	P
N. of patients	168	79	89	
Age, years				
≤60	103	49	54	0.858
>60	65	30	35	
Sex				
Male	57	26	31	0.793
Famale	111	53	58	
Smoking status				
Ever	59	26	33	0.572
Never	109	53	56	
KPS score				
>80	113	48	65	0.091
≤80	55	31	24	
TNM stage				
IIIA	154	74	80	0.376
IIIB	14	5	9	
Pathological type				
MPP	99	49	50	0.737
Non-MPP	62	29	33	
Unknown	7	1	6	
First-line treatment				
TKI	31	18	13	0.167
Non-TKI	131	58	73	
Unknown	6	3	3	
First-line treatment				
Thoracic RT	21	11	10	0.568
Non-Thoracic RT	140	64	76	
Unknown	7	4	3	
TKI				
Yes	58	32	26	0.124
No	110	47	63	
Thoracic RT				
Yes	30	13	17	0.655
No	138	66	72	

**Figure 2:**
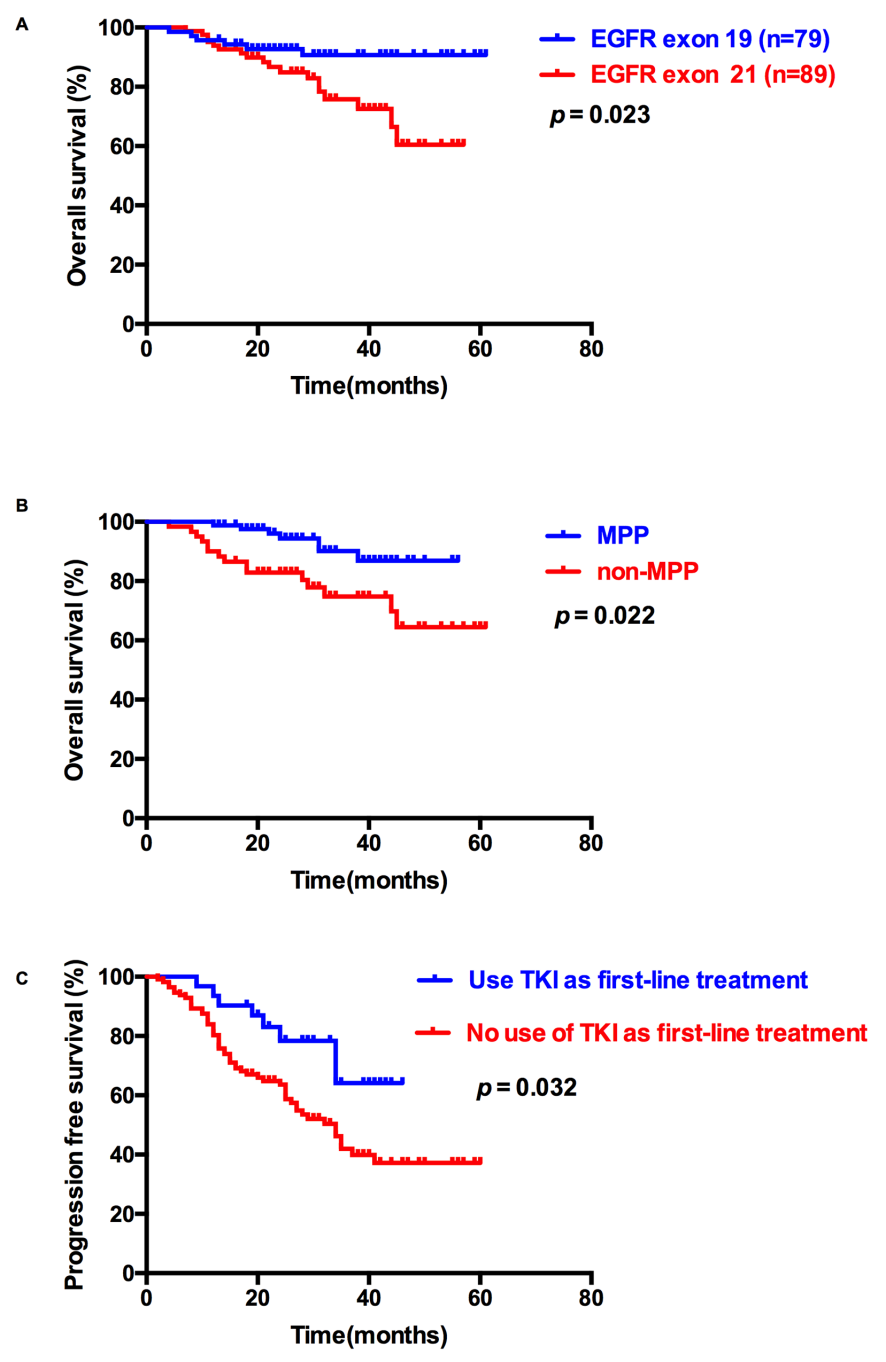
Overall survival (OS) and progression-free survival (PFS) of patients with classic EGFR mutations **(A)** Kaplan–Meier survival curves for OS analyses between EGFR exon 19 and 21 mutations. **(B)** Kaplan–Meier survival curves for OS analyses between MPP and non-MPP. **(C)** Kaplan–Meier survival curves for PFS analyses between TKI and non-TKI as first-line treatment.

Among all 168 patients with EGFR mutations, EGFR status (p=0.023), KPS score (p<0.001), and pathological type (p<0.001) were significantly associated with OS; KPS score (p<0.001) and first-line treatment (p=0.032) were significantly correlated with worse progression-free survival (PFS). In multivariate analysis incorporating EGFR status, KPS score, and pathological type, EGFR status (hazard ratio=1.681, 95% confidence interval: 1.075–2.629, p=0.023), KPS score (hazard ratio=0.053, 95% confidence interval: 0.018–0.157, p<0.001), and pathological type (hazard ratio=0.357, 95% confidence interval: 0.148–0.860, p=0.022) were the independent predictors for OS. In multivariate analysis incorporating KPS score and first-line treatment, KPS score (hazard ratio=0.148, 95% confidence interval: 0.087–0.253, p<0.001), and first-line treatment (hazard ratio=0.442, 95% confidence interval: 0.210–0.931, p=0.032) were the independent predictors for PFS (Figure 2, Tables 2 and 3).

**Table 2:** Overall survival analysis of the whole 168 patients

	Univariate analysis		Multivariate analysis	
	P	HR(95% CI)	P	HR(95% CI)
EGFR				
Exon 19	0.046	1.00	0.023	1.00
Exon 21		1.562(1.009-2.417)		1.681(1.075-2.629)
Age				
≤60	0.966	1.00		
>60		1.018(0.456-2.272)		
Sex				
Male	0.782	1.00		
Famale		0.888(0.383-2.059)		
Smoking status				
Never	0.381	1.00		
Ever		1.431(0.642-3.190)		
KPS score				
≤80	<0.001	1.00	<0.001	1.00
>80		0.044(0.015-0.128)		0.053(0.018-0.157)
TNM stage				
IIIA	0.834	1.00		
IIIB		0.857(0.202-3.638)		
Pathological type				
Non-MPP	0.025	1.00	0.022	1.00
MPP		0.372(0.157-0.881)		0.357(0.148-0.860)
TKI				
Yes	0.847	1.00		
No		1.082(0.486-2.411)		
Thoratic RT				
Yes	0.068	1.00		
No		0.399(0.149-1.069)		

Abbreviations: OS: overall survival; HR: hazard radio; MPP: micropapillary pattern; RT: radiotherapy; TKI: Tyrosine Kinase Inhibitor.

**Table 3:** Progression free survival analysis of the whole 168 patients

	Univariate analysis		Multivariate analysis	
	P	HR(95% CI)	P	HR(95% CI)
EGFR				
Exon 19	0.531	1.00		
Exon 21		1.082(0.845-1.386)		
Age				
≤60	0.393	1.00		
>60		1.252(0.747-2.099)		
Sex				
Male	0.969	1.00		
Famale		0.990(0.583-1.679)		
Smoking status				
Ever	0.762	1.00		
Never		0.921(0.543-1.564)		
KPS score				
>80	<0.001	1.00	<0.001	1.00
≤80		0.147(0.087-0.249)		0.148(0.087-0.253)
TNM stage				
IIIA	0.194	1.00		
IIIB		0.464(0.145-1.479)		
Pathological type				
MPP	0.477	1.00		
Non-MPP		0.831(0.498-1.386)		
First-line treatment				
Non-TKI	0.021	1.00	0. 032	1.00
TKI		0.418(0.199-0.848)		0.442(0.210-0.931)
First-line treatment				
RT	0.759	1.00		
Non-RT		0.884(0.403-1.940)		

Abbreviations: PFS: progression free survival; HR: hazard radio; MPP: micropapillary pattern; RT: radiotherapy; TKI: Tyrosine Kinase Inhibitor.

**Figure 3:**
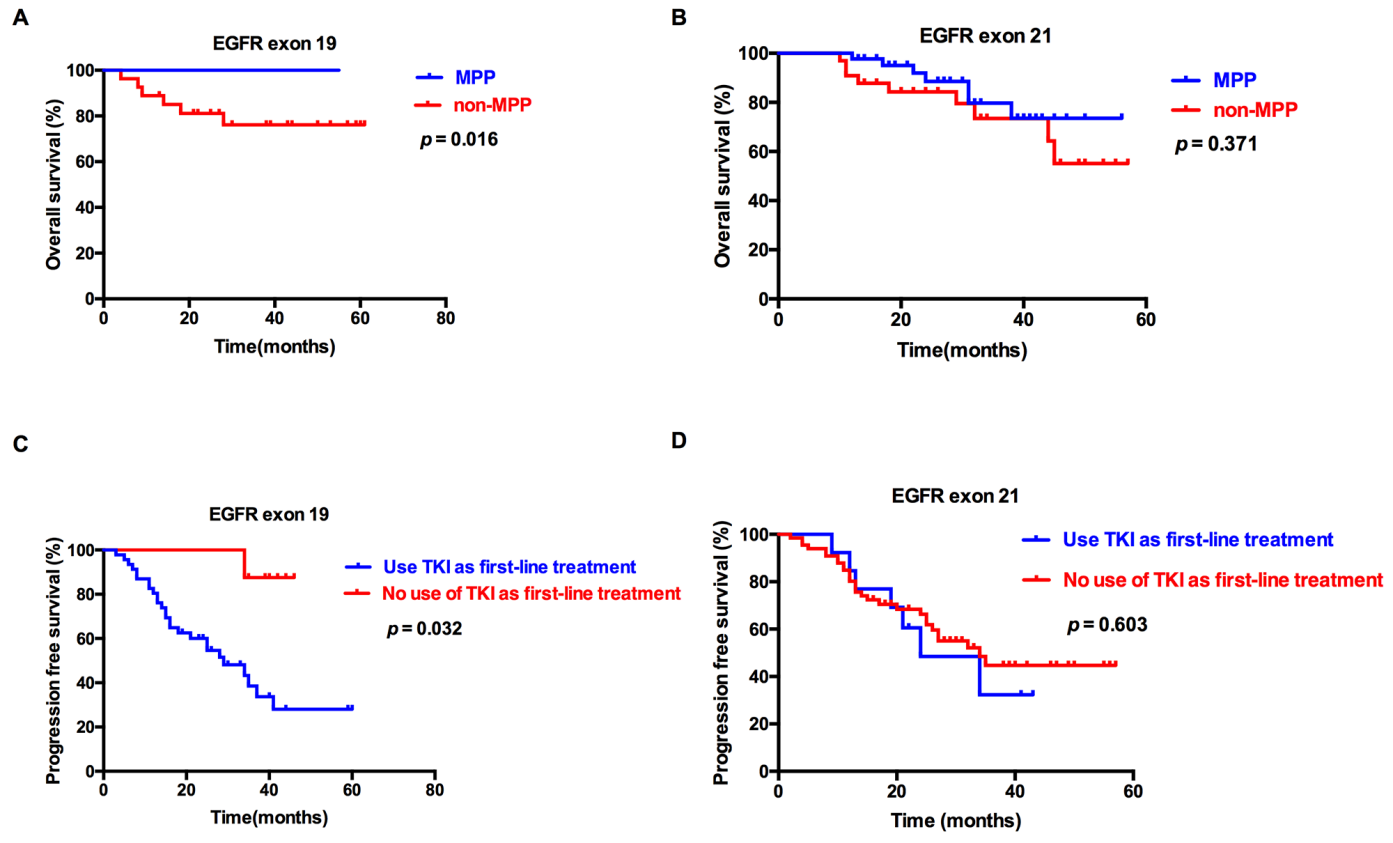
Overall survival (OS) and progression-free survival (PFS) of patients with EGFR exon 19 and 21mutations Kaplan–Meier survival curves for OS between MPP and non-MPP for patients with EGFR exon 19 **(A)** and EGFR exon 21 **(B)** mutations. Kaplan–Meier survival curves for PFS between TKI and non-TKI as first-line treatment for patients with EGFR exon 19 **(C)** and EGFR exon 21 (D) mutations.

The results demonstrated that the patients carrying exon 19 mutation had a better OS than those carrying exon 21 mutation; thus, we divided the patients into 2 subgroups: patients with EGFR exon 19 mutation and patients with EGFR exon 21 mutation. For patients with EGFR exon 19 mutation, treatment (thoracic radiotherapy or not, p=0.045), and pathological type (p=0.016) were significantly associated with OS; KPS score (p<0.001) and first-line treatment (TKI or not, p=0.008) were significantly associated with PFS. In multivariate analysis incorporating treatment (thoracic radiotherapy or not) and pathological type, pathological type (hazard ratio=0.073, 95% confidence interval: 0.009–0.611, p=0.016) was the independent predictors of OS. In multivariate analysis incorporating KPS score and first-line treatment (TKI or not), KPS score (hazard ratio=0.120, 95% confidence interval: 0.047–0.307, p<0.001) and first-line treatment (TKI or not, hazard ratio=0.109, 95% confidence interval: 0.014-0.828, p=0.032) were the independent predictors of OS (Tables 4 and 5). For patients with EGFR exon 21 mutation, KPS score (p<0.001) and treatment (TKI or not, p=0.025) were significantly associated with OS, and KPS score (p<0.001) was significantly associated with PFS. In multivariate analysis incorporating KPS score and treatment (TKI or not), KPS score (hazard ratio=0.067, 95% confidence interval: 0.022–0.207, p<0.001) was the independent predictor of OS (Figure 3 and Supplementary Figure 1, Tables 4 and 5).

**Table 4:** Overall survival analysis of patients with exon 19 mutation and exon 21 mutation seperately

	EGFR exon 19				EGFR exon 21			
	Univariate analysis		Multivariate analysis		Univariate analysis		Multivariate analysis	
	P	HR(95% CI)	P	HR(95% CI)	P	HR(95% CI)	P	HR(95% CI)
Age								
≤60	0.635	1.00			0.528	1.00		
>60		1.437 (0.321-6.420)				0.733 (0.279-1.925)		
Sex								
Male	0.496	1.00			0.966	1.00		
Famale		0.594 (0.133-2.658)				0.997 (0.354-2.810)		
Smoking status								
Never	0.503	1.00			0.556	1.00		
Ever		1.669 (0.373-7.459)				1.332 (0.513-3.454)		
KPS score								
≤80	0.181	1.00			<0.001	1.00	<0.001	1.00
>80		0.002 (0.000-17.332)				0.062 (0.020-0.190)		0.067 (0.022-0.207)
TNM stage								
IIIA	0.655	1.00			0.783	1.00		
IIIB		0.045 (0-36089)				0.813 (0.186-3.545)		
Pathological type								
Non-MPP	0.032	1.00	0.016	1.00	0.371	1.00		
MPP		0.099 (0.012-0.822)		0.073 (0.009-0.611)		0.634 (0.234-1.720)		
TKI								
Yes	0.123	1.00			0.025	1.00	0.103	1.00
No		0.189 (0.023-1.573)				3.032 (1.146-8.022)		2.265 (0.847-6.056)
RT								
Yes	0.045	1.00	0.059	1.00	0.357	1.00		
No		0.216 (0.048-0.969)		0.128 (0.015-1.081)		0.497 (0.112-2.202)		

Abbreviations: OS: overall survival; HR: hazard radio; MPP: micropapillary pattern; RT: radiotherapy; TKI: Tyrosine Kinase Inhibitor.

**Table 5:** Progression free survival analysis of patients with exon 19 mutation and exon 21 mutation seperately

	EGFR exon 19				EGFR exon 21			
	Univariate analysis		Multivariate analysis		Univariate analysis		Multivariate analysis	
	P	HR(95% CI)	P	HR(95% CI)	P	HR(95% CI)	P	HR(95% CI)
Age								
≤60	0.722	1.00			0.130	1.00		
>60		1.152 (0.529-2.508)				0.584 (0.292-1.172)		
Sex								
Male	0.959	1.00			0.935	1.00		
Famale		0.980 (0.452-2.126)				0.970 (0.466-2.019)		
Smoking status								
Ever	0.731	1.00			0.467	1.00		
Never		1.145 (0.528-2.484)				0.762 (0.367-1.584)		
KPS score								
>80	<0.001	1.00	<0.001	1.00	<0.001	1.00		
≤80		0.082 (0.032-0.211)		0.120 (0.047-0.307)		0.208 (0.106-0.410)		
TNM stage								
IIIA	0.651	1.00			0.201	1.00		
IIIB		0.629 (0.084-4.711)				0.394 (0.095-1.643)		
Pathological type								
Non-MPP	0.324	1.00			0.979	1.00		
MPP		0.682 (0.319-1.459)				0.991 (0.492-1.991)		
First-line treatment								
Non-TKI	0.008	1.00	0.032	1.00	0.603	1.00		
TKI		0.067 (0.009-0.492)		0.109 (0.014-0.828)		1.246 (0.544-2.854)		
First-line treatment								
RT	0.585	1.00			0.889	1.00		
Non-RT		0.716 (0.216-2.377)				1.077 (0.380-3.053)		

Abbreviations: PFS: progression free survival; HR: hazard radio; MPP: micropapillary pattern; RT: radiotherapy; TKI: Tyrosine Kinase Inhibitor.

**Figure 4:**
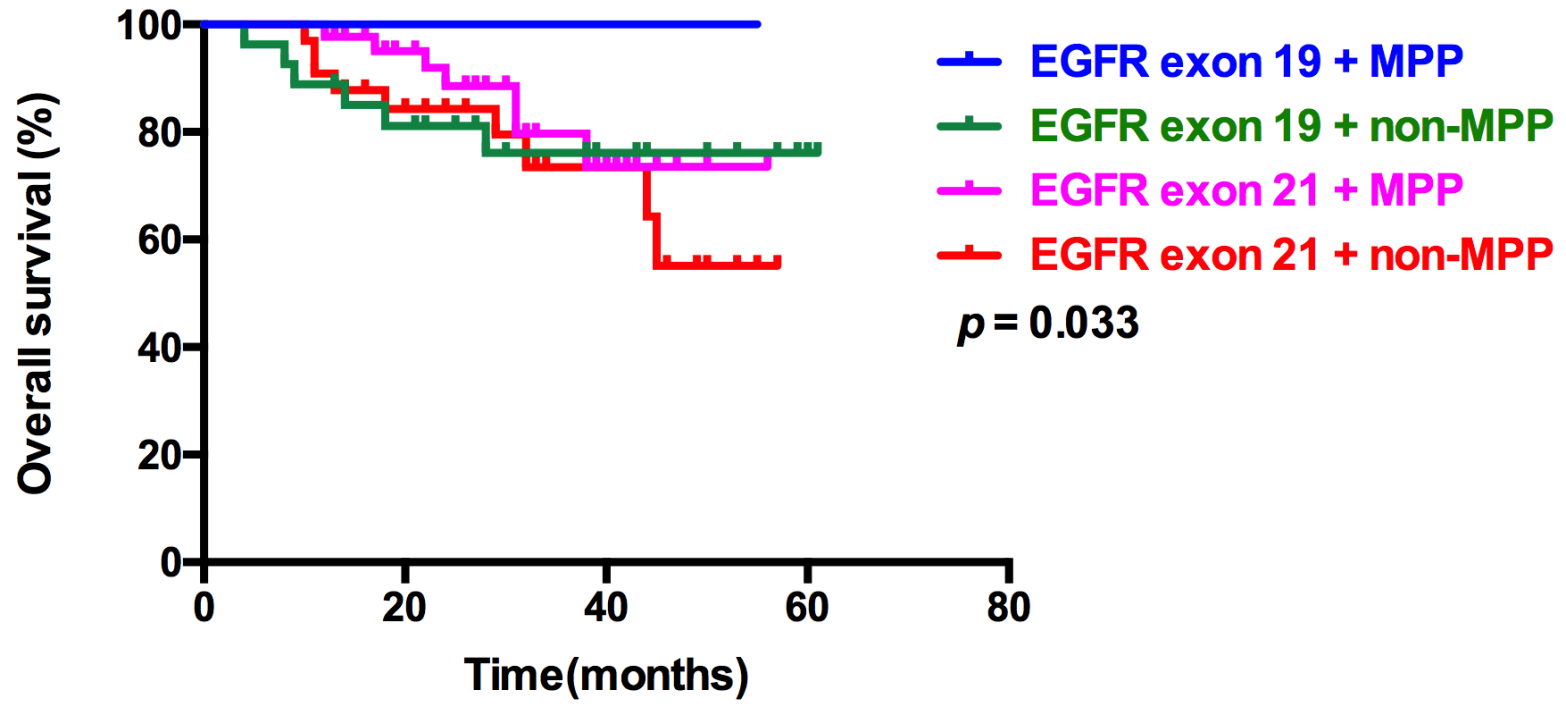
Kaplan–Meier survival curves for OS among 4 subgroups, based on EGFR mutation types and pathological types Kaplan–Meier survival curves for OS among 4 subgroups: EGFR exon 19 mutation and MPP, EGFR exon 19 mutation and non-MPP, EGFR exon 21 mutation and MPP, and EGFR exon 21 mutation and non-MPP.

We then separated the 168 patients into 4 subgroups: patients with EGFR exon 19 mutation and showing MPP in pathological type, patients with EGFR exon 19 mutation and showing non-MPP in pathological type, patients with EGFR exon 21 mutation and MPP in pathological type, and patients with EGFR exon 21 mutation and non-MPP in pathological type. Notably, patients with EGFR exon 19 mutation and MPP in pathological type had the longest OS (53.72 months), whereas patients with EGFR exon 21 mutation and non-MPP in pathological type had the shortest OS (44.9 months, p=0.033, Table 6 and Figure 4). This result demonstrated that lung adenocarcinoma with EGFR exon 19 mutation and MPP in pathological type may be good prognostic factors for OS.

**Table 6:** Overall survival analysis of four subgroups

Group	OS (months)	SD	95% CI
EGFR exon 19 + MPP	53.72	1.26	51.24-56.20
EGFR exon 19 + non-MPP	49.85	4.02	41.96-57.73
EGFR exon 21 + MPP	48.50	2.50	43.60-53.40
EGFR exon 21 + non-MPP	44.90	3.29	38.45-51.36

Abbreviations: MPP: micropapillary pattern.

## DISCUSSION

In our study, survival analysis results indicated significant differences in OS between EGFR exon 19 mutation and EGFR exon 21 mutation. Comparison of the Kaplan–Meier curves suggested that the OS of patients with exon 19 mutation was longer than those with exon 21 mutation. However, no significant differences in PFS were indicated between patients with exon 19 mutation and those with exon 21 mutation. Our results indicated that exon 19 mutation may be an indicator of good prognosis compared with exon 21 mutation, which is similar to previous reports [12]. Further investigations are required to address these differences.

As a unique pathological morphology, lung adenocarcinoma with a micropapillary pattern (MPPAC) has drawn increased attention from researchers in recent years. The cells of MPPAC are small, cube-shaped, budding with clustered growth, and lack fibrovascular development. Researchers have discovered that the structure appears in various tumors, such as breast cancer [13], bladder cancer [14], colorectal cancer [15], and ovarian cancer [16]. According to published studies, MPPAC most commonly occur in males and nonsmokers and is associated with lymphatic invasion, pleural invasion, and lymph node metastases [13, 17].

MPP has been verified to be an unfavorable prognostic marker in early-stage lung adenocarcinoma egardless of cohorts [18–21]. However, the role of MPP with regard to prognosis remains inconclusive in advanced-stage lung adenocarcinoma. Zhang et al. have reported that MPA (5% of MPP) had statistically worse recurrence-free survival, compared with nonmicropapillary-predominant adenocarcinoma with MPP observed in 5% of stage I patients; a similar correlation was not found in stage II–III patients [22]. Campos-Parra et al. have indicated that high-grade adenocarcinoma (micropapillary-, papillary-, and solid-predominant) is associated with better survival, compared with intermediate-grade adenocarcinoma (lepidic- and acinar-predominant) in advanced adenocarcinoma (stages IIIB and IV); the median PFS and OS were 6.4 vs. 5.5 months (p= 0.009) and 25 vs. 16.8 months (p= 0.023), respectively. For this result, they considered that a better response to chemotherapy probably contributed to this phenomenon [23]. Subsequently, Clay et al. have shown that MPP is not a predictor of unfavorable survival in stage III–IV [24]. By contrast, Warth A et al. have indicated that the presentation of MPP is a predictor of unfavorable outcome in not only early-stage adenocarcinoma but late-stage adenocarcinoma as well [25].

Previous studies have investigated the prognostic value of lung adenocarcinoma with an MPP, compared with those without such a pattern, or micropapillary-predominant lung adenocarcinoma compared with other histologic subtypes. However, as far as we were concerned, this study represents the first comparison study between MPP-positive adenocarcinoma and MPP-negative adenocarcinoma in EGFR exon 19 and 21 mutations of TNM stage III lung adenocarcinoma with regard to clinicopathologic characteristics and prognosis.

In the current study, we included patients with stage III lung adenocarcinoma who received radical surgery harboring EGFR exon 19 or 21. Notably, MPP was a good prognosis predictor for patients with EGFR exon 19 mutation, which varies from the results previous reported. We further divided the patients into 4 subgroups according to EGFR mutation types and pathological types; patients with exon 19 mutation and MPP had the longest OS, while those with exon 21 mutation and MPP negative had the worst OS. The differences may be attributed to better response to and better PFS from chemotherapy (61.76% vs. 37.5%) and TKIs (86.67% vs. 75%); this finding is similar to the results in a previous study in which patients with MPP harboring EGFR mutations had better survival when they received TKI treatment, compared with those with either no treatment [22].

The current study includes several limitations. First, the finding that patients with MPP pathological type had a significantly worse OS than those without MPP pathological type was based on a small number of patients and thus needs to be validated in a larger study. Second, we used cDNA-PCR sequencing as the experimental method to identify mutations; results might change if more sensitive methods are used.

In conclusion, our data analyzed risk factors of TNM stage III lung adenocarcinoma with EGFR mutations in exon 19 or 21 after radical surgery. Our results demonstrated that patients with exon 19 mutation had a better OS and were more sensitive to EGFR–TKI than those with exon 21 mutation. For patients with exon 19 mutation, the MPP pathological type may indicate good prognosis. These results may be useful in the treatment of patients with classic EGFR exon mutations.

## MATERIALS AND METHODS

### Patients and samples

From January 2011 to December 2014, we consecutively collected lung tumors resected in the Department of Pulmonary Surgery at Tianjin Medical University in Tianjin, China. Inclusion criteria for this study were as follows: (1) Patients underwent complete resection, and (2) Specimens were pathologically confirmed as lung adenocarcinomas with sufficient tissue for comprehensive mutational analyses. Pathologic slides were reviewed by 3 certified pathologists (Yan Qingna, Li Qi, and Sun Leina) to classify histologic subtypes of lung adenocarcinomas according to the International Association for the Study of Lung Cancer/The American Thoracic Society/The European Respiratory Society (IASLC/ATS/ERS) multidisciplinary classification system. The following clinicopathologic parameters for each patient were also collected: gender, age at diagnosis, smoking history, systemic treatment, pathological type, and TNM stage in line with the seventh edition of the lung cancer staging system. The PFS and OS of patients diagnosed from January 2011 to December 2014 were recorded based on a follow-up clinic visit or a telephone call.

### Mutational analysis

After frozen tumor specimens were dissected in TRIzol reagent (Invitrogen, Carlsbad, CA, USA), DNA and RNA were extracted per standard protocol, and the RNA was reverse-transcribed into cDNA with the use of the PrimeScript RT Reagent Kit (TaKaRa, Dalian, China). EGFR (exons 18–21) were routinely amplified by PCR using cDNA. Direct dideoxynucleotide sequencing was then performed to analyze the amplified products. The EGFR (exons 18–21)-amplified products obtained by PCR using DNA for sequencing were used to confirm the uncommon EGFR mutations.

### Statistical analysis

Pearson's χ^2^ test was used to investigate the correlations between 2 categorical variables. PFS and OS distribution was analyzed using the Kaplan–Meier method, and log-rank tests were employed for comparison of PFS or OS between 2 categories in univariate analysis. Multivariate survival analysis was conducted using Cox proportional hazards regression to identify independent prognostic factors. Data were statistically analyzed using SPSS 21.0 (Abbott Laboratories, North Chicago, IL, USA). Statistical significance was set at p < 0.05.

### Ethics statement

This study was approved by the institutional review board at Tianjin Medical University. Written informed consent was obtained from each patient to allow their biological samples to be genetically analyzed. The experimental protocol of this study was performed strictly in accordance with the guidelines.

## SUPPLEMENTARY MATERIALS FIGURES AND TABLES



## Abbreviations

NSCLC: non-small cell lung cancer
TNM: tumor-node-metastasis
MPPAC: adenocarcinoma with micropapillary pattern
MPP: micropapillary pattern
TKI: tyrosine kinase inhibitors
EGFR: Epidermal growth factor receptor
OS: overall survival
PFS: progression free survival
IASLC/ATS/ERS: International Association for the Study of Lung Cancer, the American Thoracic Society, and the European Respiratory Society
