# Supplementary material for: Is MPP a good prognostic factor in stage III lung adenocarcinoma with EGFR exon 19 mutation?
Source: Oncotarget. 2017 Mar 23;8(25):40594–605. doi: 10.18632/oncotarget.16505 (PMC5522255; doi:10.18632/oncotarget.16505)
Supplement: Supplementary file 1 [file oncotarget-08-40594-s001.pdf]

Is MPP a good prognostic factor in stage III lung adenocarcinoma with EGFR exon 19 mutation?

SUPPLEMENTARY FIGURE

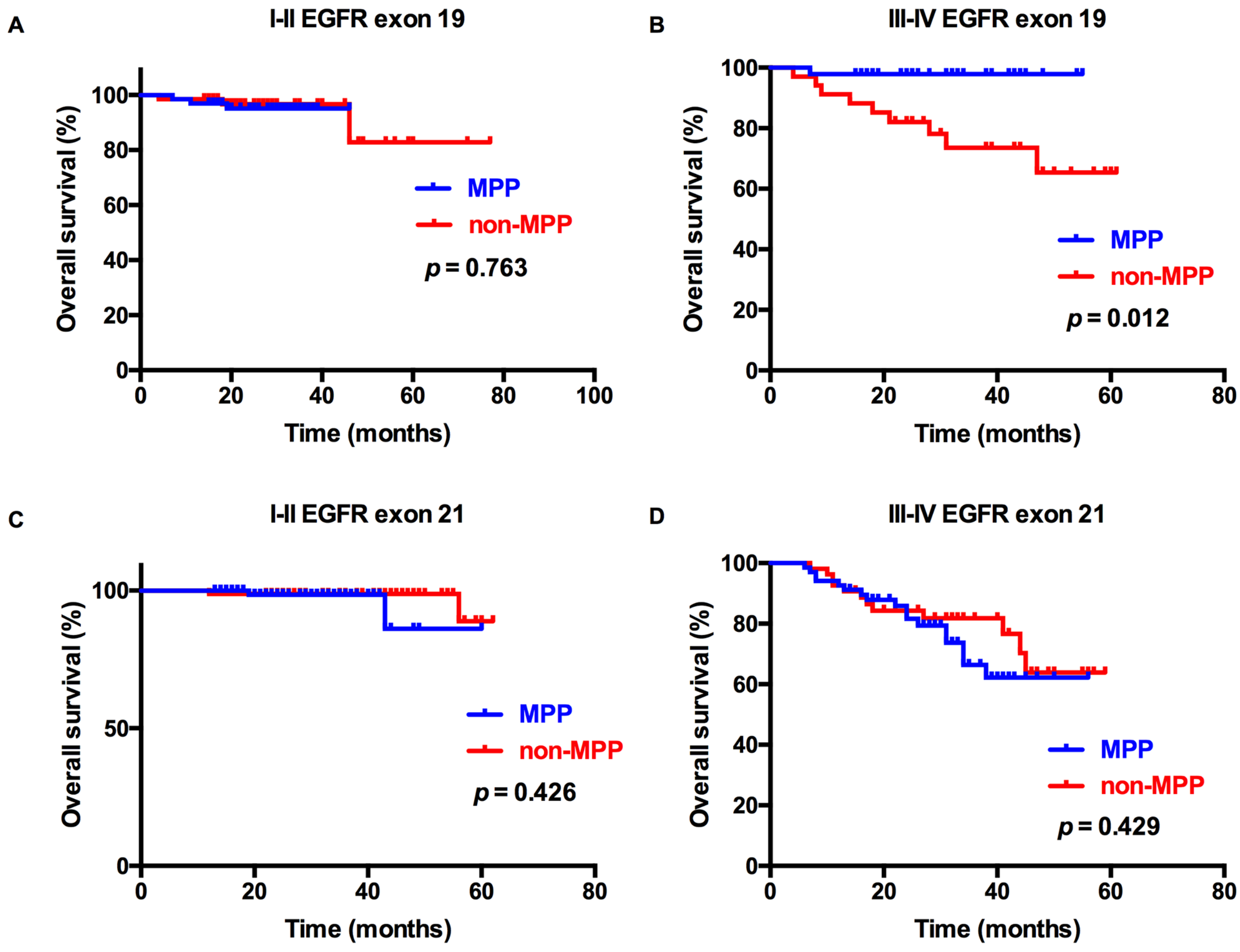

Supplementary Figure 1: Overall survival (OS) of patients with EGFR mutations in exon 19 and 21. Kaplan–Meier survival curves for OS between MPP and non-MPP in patients with TNM stage I–II **(A)** and TNM stage III–IV **(B)** lung adenocarcinoma who are harboring EGFR exon 19 mutation. Kaplan–Meier survival curves for OS between MPP and non-MPP in patients with TNM stage I–II **(C)** and TNM III–IV **(D)** lung adenocarcinoma who are harboring EGFR exon 21 mutation.
